# The immunomodulatory potential of phage therapy to treat acne: a review on bacterial lysis and immunomodulation

**DOI:** 10.7717/peerj.13553

**Published:** 2022-07-25

**Authors:** Juan Farfán, John M. Gonzalez, Martha Vives

**Affiliations:** 1 Biological Sciences Department, Faculty of Science, Universidad de Los Andes, Bogotá, Bogotá D.C., Colombia; 2 School of Medicine, Universidad de Los Andes, Bogotá, Bogotá D.C., Colombia

**Keywords:** Acne, Phage therapy, *Cutibacterium acnes*, *Propionibacterium acnes*, Immunomodulation, Bacteriophage, *Cutibacterium acnes* bacteriophages

## Abstract

**Background:**

Characterized by an inflammatory pathogenesis, acne is the most common skin disorder worldwide. Altered sebum production, abnormal proliferation of keratinocytes, and microbiota dysbiosis represented by disbalance in *Cutibacterium acnes* population structure, have a synergic effect on inflammation of acne-compromised skin. Although the role of *C. acnes* as a single factor in acne development is still under debate, it is known that skin and skin-resident immune cells recognize this bacterium and produce inflammatory markers as a result. Control of the inflammatory response is frequently the target for acne treatment, using diverse chemical or physical agents including antibiotics. However, some of these treatments have side effects that compromise patient adherence and drug safety and in the case of antibiotics, it has been reported *C. acnes* resistance to these molecules. Phage therapy is an alternative to treat antibiotic-resistant bacterial strains and have been recently proposed as an immunomodulatory therapy. Here, we explore this perspective about phage therapy for acne, considering the potential immunomodulatory role of phages.

**Methodology:**

Literature review was performed using four different databases (Europe PubMed Central-ePMC, Google Scholar, PubMed, and ScienceDirect). Articles were ordered and selected according to their year of publication, number of citations, and quartile of the publishing journal.

**Results:**

The use of lytic bacteriophages to control bacterial infections has proven its promising results, and anti-inflammatory effects have been found for some bacteriophages and phage therapy. These effects can be related to bacterial elimination or direct interaction with immune cells that result in the regulation of pro-inflammatory cytokines. Studies on *C. acnes* bacteriophages have investigated their lytic activity, genomic structure, and stability on different matrices. However, studies exploring the potential of immunomodulation of these bacteriophages are still scarce.

**Conclusions:**

*C. acnes* bacteriophages, as well as other phages, may have direct immunomodulatory effects that are yet to be fully elucidated. To our knowledge, to the date that this review was written, there are only two studies that investigate anti-inflammatory properties for *C. acnes* bacteriophages. In those studies, it has been evidenced reduction of pro-inflammatory response to *C. acnes* inoculation in mice after bacteriophage application. Nevertheless, these studies were conducted in mice, and the interaction with the immune response was not described. Phage therapy to treat acne can be a suitable therapeutic alternative to *C. acnes* control, which in turn can aid to restore the skin’s balance of microbiota. By controlling *C. acnes* colonization, *C. acnes* bacteriophages can reduce inflammatory reactions triggered by this bacterium.

## Introduction

Acne vulgaris, or acne, is considered a chronic inflammatory skin disease, which affects many people around the world, principally adolescents (Zouboulis & Bettoli, 2015). The types of acne are classified by severity, and number, and localization of lesions (Table S1) (Moradi Tuchayi et al., 2015). This skin condition compromises the pilosebaceous unit, in which the first inflammatory events occur (Dréno, 2017). During the first stages of the disease, lesions may not present redness and inflammation of local tissue. Nevertheless, there is production of inflammatory mediators in these early lesions (Antiga et al., 2015).

Acne pathogenesis is markedly influenced by four pathogenic determinants: excess sebum production, abnormal keratinization within the follicle, *Cutibacterium acnes* (formerly, *Propionibacterium acnes*) colonization in the pilosebaceous duct, and release of inflammatory molecules in the skin (Gollnick, 2015). The precise order in which these pathogenic determinants trigger the inflammatory response is not clear. In fact, acne can be triggered by an alteration of the sebaceous lipid profile (dysseborrhea), stress, irritation, cosmetics, and diet (Dréno, 2017). At molecular level, acne has several factors associated with its pathogenesis, including the action of androgens, pro-inflammatory lipids, and Pathogen-Associated Molecular Patterns (PAMPs) derived from *C. acnes* acting at different levels of the immune system (Cong et al., 2019). Keratinocytes, sebocytes, and skin-resident immune cells have receptors which are activated by these molecules. This results in the secretion of cytokines, chemokines, and other pro-inflammatory agents (Bhat, Latief & Hassan, 2017).

*C. acnes* is an anaerobic, Gram-positive bacteria, and a typical colonizer of the skin. This bacterium can interact with other cutaneous skin commensal or pathogenic microbiota (Platsidaki & Dessinioti, 2018). Resident microbiota coexists with skin immunological components and reinforce barrier functions. It is now believed that microorganisms play an important role in skin immunity, maintaining the homeostasis of immune responses, including inhibition of pathogen colonization, inflammatory reactions, and immune cell-derived responses (O’Neill & Gallo, 2018). Dysregulation of the immune response is associated with various skin diseases, including acne, in which there is a disturbance in the relationship skin microbiota and skin immune sentinels (Wang et al., 2020; Abdallah, Mijouin & Pichon, 2017). In the pathogenesis of acne, it has been suggested that the loss of the skin’s microbial diversity activates innate immunity and lead to chronic inflammation (Dréno et al., 2018). *C. acnes* can interact with other cutaneous skin commensal or pathogenic microbiota (Platsidaki & Dessinioti, 2018). Although this bacterium can be found in healthy skin (Omer, McDowell & Alexeyev, 2017), dysregulation of its growth is associated with acne. *C. acnes* strains are classified into six phylogenetic groups: IA_1_, IA_2_, IB, IC, II, and III (Dagnelie et al., 2018). It has been hypothesized that some phylotypes are commensals and contribute to skin health, while others such as phylotype IA_1_ can be associated with disease (Dagnelie et al., 2018; Dréno et al., 2018). *C. acnes* IA_1_ strains had been linked to acne given the presence of virulence factors (Cong et al., 2019; Beylot et al., 2014; Dréno et al., 2018). The effect of enriched IA_1_ isolates on skin is markedly pro-inflammatory. In contrast, a combination of different phylotypes of *C. acnes* is what leads to healthy skin (Dagnelie et al., 2019).

To treat acne several drugs has been developed and are currently prescribed. These drugs include different retinoid-derived products, antibiotics, and hormonal antiandrogens for female patients (Zouboulis & Bettoli, 2015; Latter et al., 2019; Xu & Li, 2019). These therapeutic agents are commonly prescribed because of their anti-inflammatory and anti-comedogenic activity, but they present several side effects (Aslam, Fleischer, and Feldman 2015). Oral antibiotics are also prescribed to treat acne, especially because of their anti-inflammatory effects (Farrah & Tan, 2016). However, antibiotic administration has been associated with both adverse events and bacterial resistance (Oudenhoven et al., 2015). Despite their antibacterial effect in *C. acnes* virulent strains, antibiotics are not species-specific, hence they likely promote bacterial resistance, disturbance of skin microbiota, and a growth of opportunistic pathogens (Liu et al., 2015; Karadag et al., 2020). Given the importance of skin microbiota in the regulation of immunity, an enhancement of beneficial microbial communities should be considered as part of successful acne therapy. Microbial balance has also impact in the resolution of disease flare-ups and comeback of inflammatory lesions (Woo & Sibley, 2020).

In this review, we explore the potential use of bacteriophages as a therapeutic alternative to treat acne, from the interplay between their influence on the skin’s inflammatory response and the pathogenic determinants of acne. To this end, we first examine the inflammatory nature of acne, in particular the immune response against *C. acnes*. Next, we present the available acne treatments, focusing on the anti-inflammatory properties of these drugs. Finally, we discuss the available information along with the potential of phage therapy in the treatment of acne based on its anti-inflammatory properties. Most published articles about *C. acnes* phages and the intended use of these phages for phage therapy are focused on bacterial elimination. However, the potential of immunomodulation for these phages has not been completely addressed. Here, we emphasize the inflammatory nature of the disease and the available anti-inflammatory treatment of acne, to approach phage therapy from the immunomodulation perspective. With this article, we expect to point out important gaps in the field and encourage further research directed to investigate immunomodulatory properties of *C. acnes* phages. Moreover, we intend to raise interest in researchers of other scientific disciplines, including immunologists, dermatologists, and physicians interested in new therapeutic alternatives for inflammatory and infectious diseases.

## Survey Methodology

We performed a literature search in four different databases: ePMC, Google Scholar, PubMed, and ScienceDirect, using combinations of the following keywords: “Acne”, “Acne pathology”, “Acne Therapy or Treatment”, “Antibiotic resistance”, “*Propionibacterium acnes* or *Cutibacterium acnes*”, “Inflammation or Inflammatory response”, “Immunity or Immune Response”, “Immunomodulation”, “Bacteriophage or Phage”, and “Phage therapy”. Keyword combinations were arranged according to the discussion object for each section. All relevant articles were included and summarized in a database. Following each search, a total of 372 manuscripts were screened. Articles to be included in this review were selected based on the following exclusion criteria: year of publication (2010–2020 for literature reviews, and 2015–2020 for original research articles), number of citations per year compared to other articles found, and quartile of the publishing journal (Quartiles 1 and 2). Applying selection criteria, 212 manuscripts were retrieved and assessed to be included in this review. Articles citing the literature found in our search were also explored. We included for analysis all reported outcomes of phage immunomodulation: anti- and pro-inflammatory results in phage-therapy contexts and *in vitro* assays of interactions between phages and immune system components.

## Inflammatory Response in Acne

**Figure 1:**
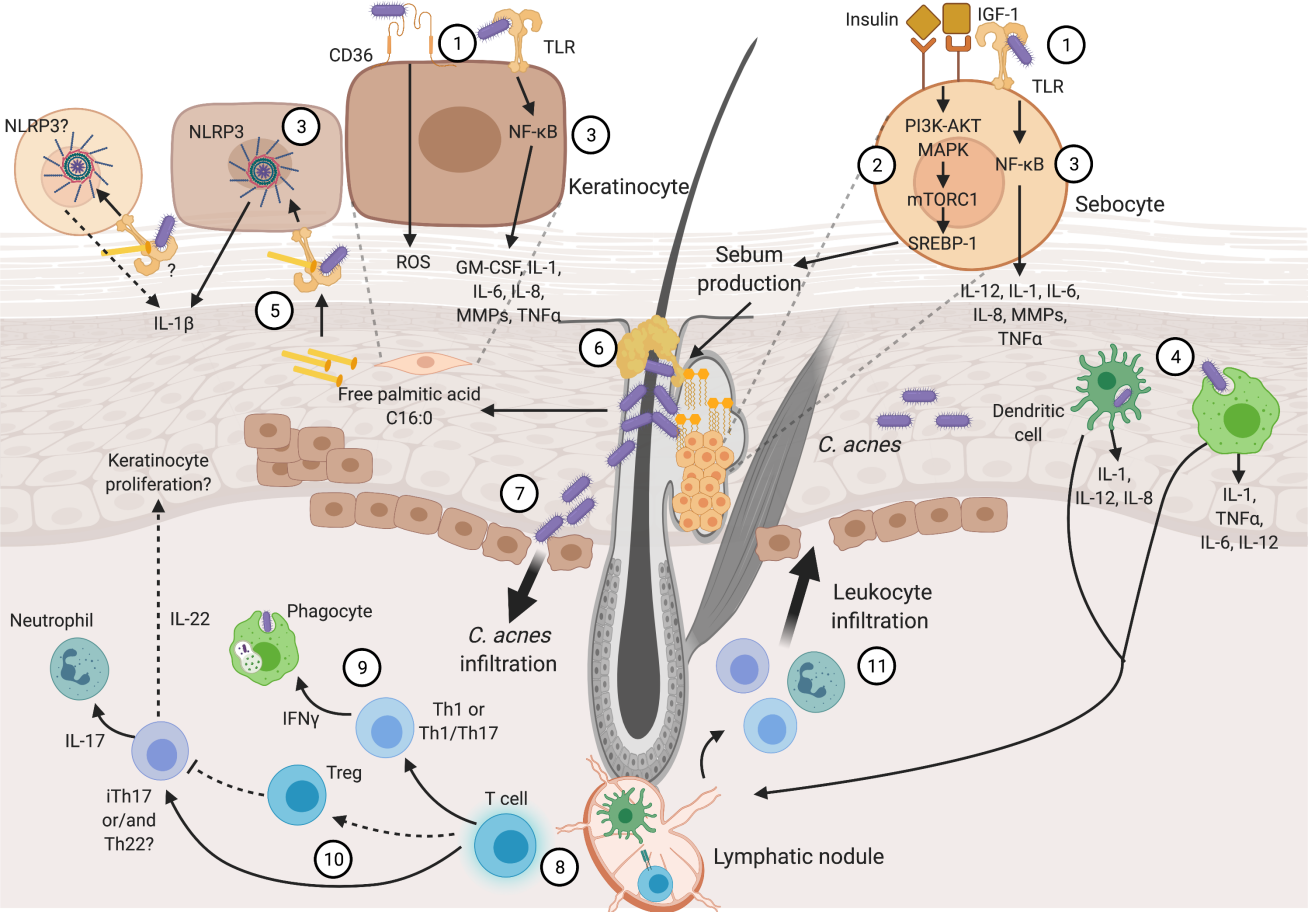
Inflammatory process in acne pathogenesis. Figure 1 (not at scale) illustrates how several pathogenic factors convey and promote inflammation in acne. Continuous narrow arrows represent events in acne pathogenesis, broad arrows show cell infiltration, grey dashed lines represent cell magnifications of sebocytes and keratinocytes, and black dashed lines represent yet unproven suggested pathways. Color conventions are blue for T cells, green for phagocytes (monocytes and macrophages) and innate immunity cells (neutrophils and skin resident dendritic cells). Insulin, IGF-1, abnormal lipid production and *C. acnes* are the main triggering factors involved in acne pathogenesis. These factors are recognized by sebocyte and keratinocyte receptors (1). Sebocytes recognize insulin and IGF-1 promoting abnormal sebum production (2). *C. acnes* is recognized in sebocytes and keratinocytes which produce pro-inflammatory cytokines through NF-κB and NLRP3 activation (3). Moreover, this bacterium can be recognized and endocyted by phagocytes and DCs in dermis and epidermis (4). Palmitic acid derived from *C. acnes* lipase activity is also recognized through through TLR2, resulting in the production of IL-1β (5). Sebum production and hyperkeratinization develop sebum plugs that favor *C. acnes* growth (6). Pro-inflammatory cytokine production and *C. acnes* outgrowth result in tissue perturbation and infiltration of the bacterium and inflammatory molecules (7). Consequently, Th cell activation and maturation occur in the dermis (8). Different Th subpopulations are related to acne pathogenesis: Th1 or Th1/Th17 stimulate phagocytes (9), Treg might not be capable of controlling iTh17 and/or Th22 that activate neutrophils and likely promote keratinocyte proliferation (10). Finally, stimulated leucocytes infiltrate through damaged tissue and promote further inflammatory reactions (11). Details in cytokine production and immune cell stimulation by acne pathogenic factors are described through the text and depicted in this figure. Abbreviations: NF-κB (Nuclear Factor kappa B), ROS (Reactive Oxygen Species), PI3 (Phosphoinositide 3-Kinase), AKT (Protein Kinase B), (MAPK) Mitogen-Activated Protein Kinase, mTORC1 (Mammalian Target of Rapamycin Complex 1), SREBP-1 (Sterol Regulatory Element-Binding Protein 1), Th (T helper cells), Treg (T Regulatory cells), iTh (Inflammatory T helper cells), TNFa (Tumor Necrosis Factor a), DC (Dendritic Cell). Figure created with Biorender.com.

Inflammation is the fingerprint of acne pathogenesis, driven by Insulin-like Growth Factor (IGF-1), altered sebum production, and *C. acnes*. These are the main factors involved in the beginning of the disease, though it is not clear which of them appears first. Then, hyperkeratinization and altered sebum production appear as typical signs of the disease. In addition, hyperkeratinization produces anoxic conditions that confers an optimal environment for *C. acnes* proliferation (Cong et al., 2019). It has been proposed that early production of inflammatory cytokines is present in sub-clinical lesions, followed by disturbed lipid production and consequent *C. acnes* propagation (Quanico et al., 2017). Various pro-inflammatory cytokines and chemokines promote chronic inflammation and disease evolution. Later, chronic inflammation in acne can include innate immune cells and CD4+ helper T cells (Th cells) (Mattii et al., 2018; Kistowska et al., 2015). Figure 1 provides an overview of the acne pathogenesis, focusing on the inflammatory nature of the disease, and its details are covered in the following lines.

### Initial inflammatory response

The acne pathogenic process is different in sebocytes and keratinocytes, as has been reviewed by Cong and colleagues (Cong et al., 2019). Both cell types, located in sebaceous glands and in the epidermis, express inflammatory markers. In keratinocytes, recognition of *C. acnes* by Toll-like Receptors 2 or 4 (TLR-2, TLR-4), or CD36 is plausibly a sufficient stimulus to produce inflammatory cytokines. Whereas, in sebocytes TLR-2 is the receptor involved in *C. acnes* sensing (Cong et al., 2019). Moreover, phagocytes and dendritic cells (DCs) can recognize and phagocyte *C. acnes* at epidermis and dermis (Mattii et al., 2018; Qin et al., 2014).

The skin homeostasis is likely maintained via a Nuclear Factor kappa B (NF-κB) negative regulator known as TNFAIP3-interacting protein 1 (TNIP1) (Erdei et al., 2018). Once *C. acnes* PAMPs are sensed through TLR-2/TLR-4, following NF-κB activation, pro-inflammatory cytokines are released (Cong et al., 2019). Thus, cytokines orchestrating the immune response in acne includes Interleukin 1β (IL-1β), Interleukin 6 (IL-6), Interleukin 8 (IL-8), and Tumor Necrosis Factor alpha (TNFα) involved in the inflammatory pathogenesis (Kim et al., 2017). *C. acnes* also induces the production of Reactive Oxygen Species (ROS) in keratinocytes through CD36 receptor recognition (Grange et al., 2009). Another inflammatory set of sensory molecules derives from the inflammasomes, which are intracellular multiprotein complexes involved in the innate immune response (De Sá & Festa Neto, 2016). Inflammasomes are activated by stimuli such as pathogens, extracellular adenosine triphosphate (ATP), and ROS. In response, inflammasomes activate caspase-1, mediating the maturation of pro-inflammatory cytokines: IL-1β, and interleukin 18 (IL-18) (Yin et al., 2015). Among the multiple subtypes of family proteins, NLRP3 has been associated with acne; *C. acnes* activates this inflammasome inducing IL-1β production in a caspase-1 dependent manner involving potassium efflux (Wang et al., 2020). This finding supports the fact that *C. acnes* is recognized by superficial receptors located in the cell membrane, rather than by intracellular receptors (Qin et al., 2014).

Recent investigations have revealed new receptors and transcription factors involved in acne pathogenesis, having important effects on hyperkeratinization, skin cell differentiation, sebum production and other inflammatory events (Oulès et al., 2020). Hyperkeratinization of the infundibulum (upper portion of the follicle) is related to diminished expression of the transcription factor GATA-6, an important modulator of keratinocyte proliferation and differentiation. When GATA-6 is down-regulated, it can conduce to disturbed immune responses in sebocytes. As a consequence of GATA-6 down-regulation in these cells, there is lessened production of Programmed Death-ligand 1 (PD-L1) and Interleukin 10 (IL-10), resulting in the diminishing of anti-inflammatory activities of these molecules (Oulès et al., 2020). One study found that altered sebum production and inflammatory mediators in sebocytes are linked to a reduced expression of the peroxisome proliferator-activated receptor γ (PPARγ), which plays a role in differentiation and anti-inflammatory response. Sebocytes may also be more insulin and IGF-1 sensitive, due to the increased expression of hormone receptors (Briganti et al., 2020). The Janus kinase/signal transducer and activator of transcription (JAK/STAT), in particular JAK1 and JAK3 proteins, and Caveolin-1 have been found to be activated in acne patients and possibly play important roles in the pathogenesis of the disease (Awad et al., 2020; Kruglikov & Scherer, 2020).

### Role of hormonal dysregulation and lipid production

Hormone dysregulation significantly affects the proliferation and differentiation of sebocytes and keratinocytes, which are related to aberrant response to androgens and insulin (Briganti et al., 2020). In keratinocytes, abnormal sebum and androgens promote keratosis (Cong et al., 2019). High glycemic and dairy diets are likely associated with high IGF-1 levels in serum, as has been found in acne affected individuals (Agamia et al., 2016). IGF-1 can up-regulate Sterol Regulatory Element-Binding Proteins (SREBPs) and Phosphatidylinositol 3-Kinase (PI3KCA) expression, which are related to sebum production by sebocytes (Kim et al., 2017). SREBPs and PI3KCA are activated via Forkhead box transcription factor (Fox) O1 expression and the mammalian target of rapamycin (mTOR) that are increased in acne affected individuals. This is also related to high-glycemic diets (Agamia et al., 2016).

Abnormal lipid presence and keratinization, derived from anomalous action of sebum production and androgens, also impact the inflammatory status in acne-affected skin. Aberrant expression of fatty acid synthase and mature sterol response element-binding protein-1 (SREBP-1) have been related to repressed Interleukin 4 (IL-4) and the anti-inflammatory cytokine IL-10, in combination with augmented Interferon γ (IFN γ). This can affect sebocytes, potentiating abnormal lipid production (Shin et al., 2019). Moreover, given its capacity to metabolize lipids by excreting lipases, *C. acnes* likely survives and thrives in sebum-rich comedones (Josse et al., 2020). Moreover, short-chain fatty acids generated by *C. acnes* lipolysis enhances pro-inflammatory cytokine secretion by inhibition of histone deacetylase activity (Sanford et al., 2019).

### Factors that promote *C. acnes* growth

As mentioned previously, acne pathogenesis has been correlated with certain strains of *C. acnes,* in particular those belonging to the phylotype IA_1_ (Lomholt et al., 2017). The abundance of type IA_1_ strains in acne is related with a disbalance in the richness of the different phylotypes of *C. acnes* in the skin. This can be explained given the presence of virulence factors in IA_1_ strains which include lipases, proteases, hyaluronate lyase, endoglycoceramidases, neuraminidases, the Christie-Atkins-Munch-Petersen (CAMP) factor, and porphyrins, which help in colonization, promote host-tissue degradation, and stimulate inflammation (Cong et al., 2019; Beylot et al., 2014; Dréno et al., 2018). Besides, virulence factors present in IA_1_ isolates are associated with other mentioned pathological factors involved in this disease. Androgen hormone activity increases sebum production inside the pilosebaceous follicle. Given the production of lipases, *C. acnes* can metabolize lipids and grow in lipid-rich acne lesions (Rocha & Bagatin, 2018). This capacity also confers a competitive advantage for *C. acnes* against other microbial skin colonizers (O’Neill & Gallo, 2018). Inflammation is induced by secretion of the virulence factors, and skin and immune cells’ recognition of bacterial PAMPs, triggering the activation of innate immune cells. Porphyrins produced by isolates belonging to the IA_1_ phylotype induces perturbations on keratinocyte membrane integrity, resulting in IL-1β production (Spittaels et al., 2021). Other virulence factors may play relevant roles in acne pathogenesis, although their exact role is not fully understood. For instance, CAMP blockage has been found effective in controlling acne in mice (Wang et al., 2018a; Wang et al., 2018b).

Although IA_1_ *C. acnes* strains are consensually stated as acne pathogenic strains, further investigation should be conducted to fully conclude this, given that other phylotypes can also harbor virulence factors. For instance, CAMP factor has been found in IA_1_, IB, and II *C. acnes* strains. Although strains different from IA_1_ are not typically related to acne, CAMP factor belonging to IB and II phylotypes can induce intense production of CXCL8 (IL-8) in response to TLR-2 recognition of this virulence factor by keratinocytes (Lheure et al., 2016).

### Tissue disruption and T cell involvement

*C. acnes* overgrowth and pro-inflammatory cytokines provoke a continuous inflammatory response and, eventually, cause tissue rupture and infiltration of *C. acnes* and inflammatory molecules into the dermis (Mattii et al., 2018). Thus, the initial immune effector response can be followed by the adaptative immune response to *C. acnes*, mainly driven by CD4+ T cells (Th) (Cong et al., 2019). The adaptative immune response is mediated by specialized Th1 cells, that can be self-maintained independently in absence of *C. acnes* (Antiga et al., 2015). Specialized branches of skin immunity are activated by chemokines that work as chemoattractant factors that expand immune reactions (Griffith, Sokol & Luster, 2014). The chemokine CXCL1 (Keratinocyte derived chemokine-KC) is upregulated in acne, acting as neutrophil chemoattractant, and CXCL2 (SDF-1)—produced by macrophages—is also upregulated and operates to attract polymorphonuclear leukocytes (Li et al., 2019). Also, sebocytes can produce IL-8 in response to acne inflammatory determinants, attracting neutrophils, monocytes, and T cells (Mattii et al., 2018).

Bacterial infiltration activates T cells, and activated Th cells are stimulated with IL-1β (from keratinocytes and sebocytes) and IL-12 to be further specialized into IFN-γ producing Th1 or Th1/Th17 cells, and IL-17 and IL-22 producing iTh17 cells (Sallusto, 2016). In particular, Th17 differentiation cytokines are highly expressed in acne (IL-1β, IL-6, Transforming Growth Factor β-TGF-β-, and IL23p19) (Kelhälä et al., 2014). IL-17/Th17 pathways have been described in acne, as a result of *C. acnes* sensing by immune cells (such as peripheral blood mononuclear cells) that promote differentiation of Th cells into Th17 cells (Agak et al., 2014). However, it has been reported that Th17 clones specific to pathogenic *C. acnes* strains are not fully competent to eliminate this bacterium, ironically contributing to acne pathogenesis. Th17 cells produce IL-17 that attracts neutrophils, which in turn prolong inflammation (Agak et al., 2018). Sebocytes also promote T cell differentiation into Th17 cells, via IL-6, TGF-β and IL-1β production that are released during acne development. These Th17 cells are particularly responsive to *C. acnes*, given the interaction between this bacterium and sebocytes, which in turn, aids dendritic cell maturation. As a result, DCs preferentially present *C. acnes* antigens (Mattii et al., 2018).

As well as Th17, acne lesions also present Th1 products (IL-17 or IFN γ) and transcription factors (ROR γt or T-bet). Their presence may correspond to *C. acnes*-derived activation of immune cells that promote responses of these lymphocytes that can produce IL-17A and IFN γ, which also suggest Th17/Th1 cell activation or combined Th1 and Th17 cell presence (Kistowska et al., 2015).

Regulatory T cell (Treg) subpopulations have been suggested to play important roles in acne pathogenesis. Treg cells are lymphocytes that modulate inflammatory reactions, mainly exerted by Th17 cells (Sallusto, 2016). Identified by the transcription factor FOXP3, these cells were detected in acne-affected individuals in their epidermis and in papillary dermis. Nevertheless, these cells do not appear to be associated with IL-17-producing cells, which might suggest a deficit in Treg function in acne vulgaris to control inflammatory events (Farag et al., 2020).

More elusive is the possible function of Th22 cells in acne. IL-22 production has been reported in acne lesions, which can be produced by inflammatory Th17 cells (iTh17), as suggested by Kelhälä and collaborators (Kelhälä et al., 2014). This cytokine has been related to keratinocyte proliferation and migration (Eyerich & Eyerich, 2018). Even though IL-22 can be produced by iTh17, this cytokine is principally produced by Th22 cells (Sallusto, 2016). Therefore, Th22 cells might play a role in acne pathogenesis that is still to be unveiled, as reported in other skin inflammatory diseases (Eyerich & Eyerich, 2018).

Lymphocyte and other immune cells are further involved in defective scaring. The immune response caused by T cells, neutrophils, and macrophages is maintained in patients prone to scar formation, which also presents B cell infiltrate. Moreover, sebaceous glands are likely irreversibly damaged (Carlavan et al., 2018). Regarding Th cells, Th17/Th1 cells have been found to be elevated in abnormal scaring, as have the degeneration of elastic and collagen fibers, and diminished epidermal cell proliferation. These factors are likely modulated by abnormal TGF-β1 signaling, which alongside IL-6 induces Th17 differentiation and subsequential production of pro-inflammatory IL-17 (Moon et al., 2019). Finally, Th cell activation leads to leukocyte infiltration to epidermis, neutrophil activation by Th17 cells, and stimulation of phagocytes by Th1 or Th1/Th17 cells. Besides, iTh17 cells and/or Th22 cells produce IL-22 that might stimulate hyperkeratosis. These events support inflammation in the affected dermis of acne patients.

## Anti-inflammatory Acne Treatment

Acne treatment includes the prescription of one or more drugs that can be applied topically or systemic. The different approaches, agents, and emerging treatments have been extensively reviewed elsewhere (Thiboutot et al., 2018; Fox et al., 2016; James, Burkhart & Morrell, 2009), and are summarized in Table 1. Alternative agents have been proposed, including antimicrobial peptides, probiotics, plant extracts, and minerals (Fox et al., 2016), and more research is being undertaken to study the potential of molecules such as sarecycline, tazarotene, minocycline, clascoterone, and cannabidiol (Kircik, 2019). Therapeutic targets are diverse and the use of one or a combination of different types of therapeutic agents depend on acne severity, a patient’s medical record and gender, propensity to scarring, and the physician’s criteria (Thiboutot et al., 2018). More affected individuals are usually treated with a combination of anti-acne agents; for instance, nodular or conglobate acne is treated with a combination of oral isotretinoin (a retinoid) and antibiotics. This treatment can be coupled with anti-androgen drugs in female patients (Gollnick et al., 2016).

**Table 1:** Prescribed pharmaceutical agents to treat acne (Kapoor, Saigal & Elongavan, 2017; Moradi Tuchayi et al., 2015; Fox et al., 2016).

Therapy class	Mechanisms of action	Examples
Retinoids	-Inhibition of comedogenesis, reduction of hyperkeratosis	Isotretinoin, adapalene, motretinide, retinoil- β-glucuronide, tazarotene, tretinoin
	- Anti-inflammatory effects	
Antibiotics	-Inhibition of bacterial protein synthesis	Lincosamide: Clindamycin
	- Anti-inflammatory effects	Macrolides: erythromycin, azithromycin, roxithromycin
		Tetracyclines: doxycycline, lymecycline, minocycline
	-Inhibition of bacterial nucleic acid replication	Quinolone: levofloxacin
	-Anti-inflammatory effects	
	-Interference with bacterial metabolism and inhibition of nucleic acid synthesis	Trimethoprim-Sulfamethoxazole
	-Anti-inflammatory effects	
Hormonal	-Reduction of androgen activity	Oral contraceptives, spironolactone, flutamide
	-Reduced sebum production	
Other antimicrobial and anti-inflammatory agents	-Antimicrobial	Azelaic acid, benzoyl peroxide, chemical peels, corticosteroids, dapsone, hydrogen peroxide, niacinamide, salicylic acid, sodium sulfacetamide, sulfur, triclosan, clofazimine, corticosteroids, ibuprofen, zinc sulfate
	-Anti-inflammatory	
	-Keratolytic	
	-Comedolytic	

Retinoids are a class of drugs derived from vitamin A and one of the most widely used treatments in acne therapy; given their properties as anti-comedogenic, reducers of abnormal keratinization, and anti-inflammatory drugs (Leyden, Stein-Gold & Weiss, 2017). These molecules act by binding on two different receptors (Retinoic acid receptors; Retinoid X Receptors), located in the nucleus, exerting retinoid-inducible gene activation and repression that derives in immunomodulation by decreasing the expression of TLR-2 receptors, reduction of abnormal follicular cell differentiation, prevention of pilosebaceous obstruction, and reduction of sebum production (Chien, 2018). There are four generations of retinoid molecules developed as acne treatments (Table S1) (Chien, 2018). Oral isotretinoin belongs to the first-generation retinoid, and is the most frequently prescribed to treat severe cases of acne (Costa et al., 2018). Isotretinoin has effects on hormone regulation, immunomodulation, and even reduction of *C. acnes* colonization (Tan et al., 2016; Ryan-Kewley et al., 2017). Nevertheless, the mechanism by which isotretinoin exerts anti-microbial properties on this bacterium or other organisms of the skin microbiota is not clear (Ryan-Kewley et al., 2017).

Treatment effectiveness depends upon isotretinoin concentration, delivery vehicle, combination with other drugs, exposure time, and patient condition (Landis, 2020). However, acne treatment with oral isotretinoin and other retinoids has been associated with several side effects. Isotretinoin most frequently causes xerosis, and facial erythema. Other less frequent adverse events include psychiatric symptoms, ocular lesions, increased cholesterol, and serum triglyceride levels (Brzezinski et al., 2017). Its prescription is not recommended in pregnant women (Costa et al., 2018). One way of reducing isotretinoin-related adverse events is to diminish its therapeutic concentration and combine it with another agent or drug. However, side effects can also appear for other treatments combined with isotretinoin (Xia et al., 2018). Acne relapses can occur following isotretinoin treatment, meaning that longer periods of treatment are needed to clear inflammatory lesions. Indeed, it is recommended to prolong the treatment once the acne has cleared up, to prevent relapses. However, higher doses can increase the probability of the occurrence of adverse events (Landis, 2020).

Other oral therapies with direct or indirect anti-inflammatory properties are anti-hormonal therapies, specially prescribed for women. These include contraceptives, spironolactone, cyproterone acetate, and flutamide. These molecules impact androgen availability, androgen binding, and sebaceous gland impairment. However, side effects also may appear and can be severe, including gastric disorders, depression, menstrual irregularities, and hematological disorders (Elsaie, 2016). Other chemical agents such as azelaic and pyruvic acid interfere with *C. acnes* metabolism, reducing free fatty acids, and therefore reducing inflammation derived from abnormal lipid profiles (Chilicka et al., 2020). Besides chemical treatments, light-based therapies and microneedle radiofrequency are physical alternatives that have recorded anti-inflammatory activities reducing IL-1α and controlling inflammatory reactions from sebaceous glands, respectively (Kim et al., 2020; Li et al., 2018). However, these therapeutic agents have been related to local adverse events, in particular irritation, pain, erythema, and edema.

Antibiotics are also frequently prescribed to manage acne, namely tetracyclines, macrolides, clindamycin, and trimethoprim/sulfamethoxazole. Their use in acne therapy may be justified because of their antimicrobial properties, controlling *C. acnes* proliferation. Nevertheless, beyond bacterial elimination, these antimicrobials have anti-inflammatory properties (Bienenfeld, Nagler & Orlow, 2017). Oral tetracyclines reduce initial inflammatory responses, targeting neutrophil chemotaxis, inflammatory cytokines, metalloproteinases (MMPS), and ROS (Farrah & Tan, 2016). As a second option, macrolides can be prescribed when tetracyclines are contraindicated (Marson & Baldwin, 2019). The macrolide-class antibiotic erythromycin was one of the most frequently used antibiotics to treat acne. Nevertheless, prescription patterns of topical erythromycin changed to other antimicrobials, such as clindamycin. This change can be attributed to the emergence and spread of antibiotic resistance, since erythromycin was first introduced to the market. A similar pattern is to be expected for clindamycin and other antibiotics (Austin & Fleischer, 2017). New antibiotic molecules are arising, including sarecycline (Deeks, 2019), topical minocycline (Gold et al., 2019), the sulfone dapsone (Al-Salama & Deeks, 2017), and the quinolone-like antimicrobial VCD-004 (Ghosh et al., 2018). Although promising, these new alternatives have drawbacks: sarecycline has high activity against tetracycline-resistant strains, but less so against isolates bearing tetracycline-resistance mutations, *tet(K)* and *tet(M)* (Zhanel et al., 2019); long-term efficacy and safety studies are still lacking for minocycline foam (Gold et al., 2019); dapsone may lead to local erythema, pruritus, dryness, exfoliation, and pain (Al-Salama & Deeks, 2017); and there is already a report of *C. acnes* isolates with mutations in DNA gyrase, which is the target of VCD-004 (Nakase et al., 2016).

Due to the increasing acquisition of antibiotic-resistance mechanisms, it is important to reduce antibiotic use. As a matter of fact, no antibiotic is recommended as a monotherapy or as a long-lasting therapy (Marson & Baldwin, 2019). The first-line therapeutic option to antimicrobial resistance in acne therapy is benzoyl peroxide (BPO), which is an antimicrobial with a potent oxidative effect (Thiboutot et al., 2018). However, BPO is also considerably irritant for most patients, diminishing patient adherence to treatment (Otlewska, Baran & Batycka-Baran, 2020).

New therapeutic alternatives for acne are emerging. These new drugs are sebo-suppressive, anti-inflammatory, or comedolytic, interfering in different inflammatory pathways including Melanocortin 5 Receptor (MC5R), interleukin signaling, and leukotriene inhibitors (Trivedi et al., 2018). Superoxide dismutase 3 (SOD3) can suppress TLR-2 expression in sebocytes and keratinocytes exposed to *C. acnes* or bacterial lipopolysaccharide (LPS), resulting in NF-κB inhibition. SOD3 also reduces NLRP3 inflammasome activation. Similar results were observed *in vivo*, in a murine model (Nguyen, Sah & Kim, 2018). Plant-derived products, such as *Myrtus communis* leaf extract have anti-microbial activity against IA_1_ and clindamycin-resistant *C. acnes* strains, presumably by targeting bacterial virulence (Pécastaings et al., 2018). Designed peptides based on naturally-occurring peptides can display antimicrobial properties, that may be efficient at relatively low concentrations (Woodburn, Jaynes & Clemens, 2020). Nonetheless, large clinical studies are required to reveal its exact therapeutic mechanism and subsequent development.

Other emerging alternatives target microbiota homeostasis, an interesting approach for acne treatment, since its pathogenesis has been related to microbial imbalance (Trivedi et al., 2018). Among these, phage therapy emerges as an alternative to anti-microbial resistance without disturbing the host microbiota (Kutter et al., 2010).

## Phage Therapy and Immunomodulation

**Figure 2:**
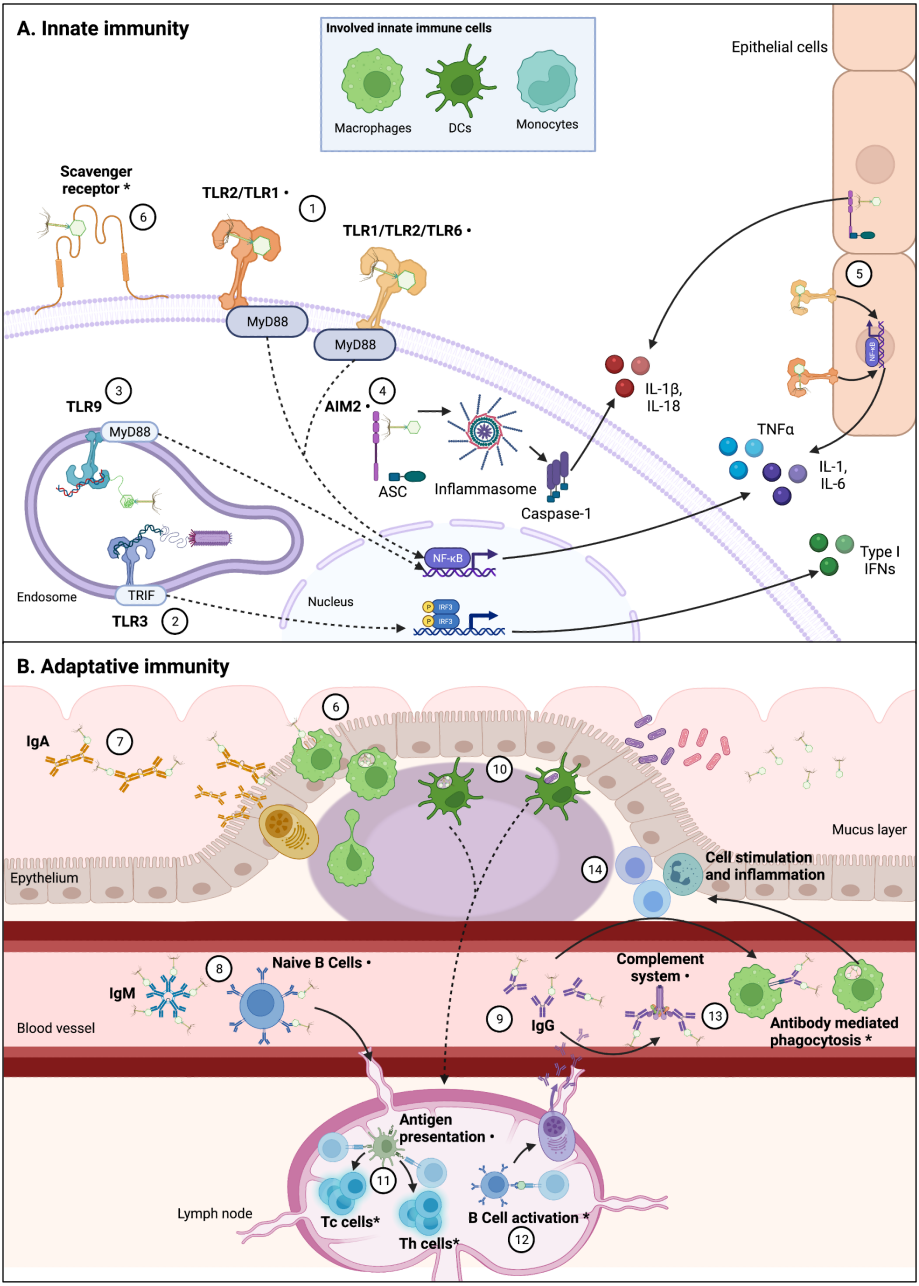
Interactions between phages and immune system components are varied and complex. Figure 2 (not at scale) show confirmed, indirectly confirmed (*), and possible interactions (⋅) between various phages and immune system components. Continuous lines represent immune pathways and dashed lines represent pathways with intermediary steps not showed in this figure. (A) Innate immune cells including macrophages, dendritic cells (DCs), and monocytes are involved in these direct interactions, likely through PRRs. Epithelial cells also present receptors that can sense PAMPs and could be involved in cytokine production derived from phage-cell interactions. (1) Receptors such as TLR-2/TLR-1 and TLR-1/TLR-2/TLR-6 can be involved in the recognition of phage particles that result in production of cytokines, via Myeloid Differentiation Primary Response 88 protein (MyD88) coupling and NF-κB activation. (2) Sweere et al. (2019) have found phagocyte-mediated endocytosis of a filamentous *Pseudomonas* phage with a subsequent production of TNFα dependent upon TLR-3 endosomal sensing of phage genome, through TIR-domain-containing adapter-inducing interferon-b (TRIF) coupling and Interferon Regulatory Factors (IRF) activation. Endocytosis of *Caudovirales* phages by DCs was also confirmed by Gogokhia et al. (2019), (3) in which TLR-9 sensing of phage genomes results in NF-kB activation. (4) Phage interactions with phagocytes that result in IL-1b and IL-18 production can be related to cytosolic phage genome detection by Absent in Melanoma 2 (AIM2) receptor recognition, via Caspase-1 activation. (5) Production of cytokines in epithelial cells can also be related to TLR and AIM2 phage detection. (6) Phagocytosis by DCs and macrophages are likely initiated by recognition of Scavenger receptors, representing the first step from an innate response to an adaptative response. The main adaptative immunity interaction with phages has been described in the characterization of antibody recognition of phages. Immunoglobulins including mucus excreted IgA (7), circulating acute-phase IgM (free or bound to naïve B cells) (8), and circulating IgG (in particular IgG2) (9) can recognize some *Caudovirales*. (10) Phagocytosis by DCs can imply antigen presentation (11) to T helper (Th) and T cytotoxic (Tc) T cells, which has been indirectly confirmed, and can be derived from phage or bacterial-derived antigens (Gogokhia et al., 2019; Fluckiger et al., 2020). (12) Specific anti-phage IgG indirectly confirms B cell activation, which derives in maturation of memory and secretory B cells. (13) Antibodies binding to phages can also result in complement system activation and antibody mediated phagocytosis, which in turn can explain increasing population of leukocytes derived from phage interaction with the immune system, and inflammation (14). As depicted in this figure, there is not a unique pathway to phage recognition by immune system components, and in various cases the complete recognition and effector pathway is yet to be unveiled. Figures created with Biorender.com.

The increasing phenomenon of antimicrobial resistance had led to propose new therapeutic alternatives (Tacconelli et al., 2017; World Health Organization, 2015). One of these alternatives is phage therapy, which consists in the use of bacteriophages (phages for short), viruses that infect bacteria, to control bacterial infections and contamination (Kutter et al., 2010). Although phage therapy is intended to kill bacteria, in recent years alternative applications have been suggested for phages due to their potential interactions with the eukaryotic cells (Górski et al., 2018). Scientific reports have showed that phages interact direct or indirectly with the mammal immune system; by infecting and eliminating bacterial hosts, phages can indirectly impact immunity, helping or improving immune responses to bacterial pathogens (Barr, Youle & Rohwer, 2013; Roach et al., 2017). However, direct interactions between phages and the immune system have also been reported (Fig. 2) (Van Belleghem et al., 2019; Sweere et al., 2019; Khan Mirzaei et al., 2016). These induced immune responses to phages trigger innate and adaptative mechanisms.

### Innate immune responses

Phage infection of host bacteria can have an indirect effect on the immune response, due its capacity to control bacterial growth or colonization. By attacking bacteria, phages favor bacterial clearance and immune responsiveness (Leung & Weitz, 2017). These outcomes may be in part due to effective bacterial number reduction, which helps the innate immunity to eradicate bacterial infections quicker and more efficiently. It has been reported that phages are necessary to eliminate an invading pathogen by reducing bacterial populations, which facilitates the function of innate immune cells (Roach et al., 2017). It was also found that certain phages may represent an alternative defense mechanism against bacteria, by inhabiting animal body niches and serving as an additional barrier to colonizers (Almeida et al., 2019). Therefore, there is a relationship between epithelial tissue, epithelial defenses, and the activity of phages in eliminating bacterial pathogens, helping to regulate local immune homeostasis (Vitetta, Vitetta & Hall, 2018). Phages can also diminish bacterial virulence by eliminating encapsulated bacterial capsules or disrupting bacterial inflammation-inducing molecules, such as LPS. As a result, bacteria become more prone to the attack of phagocytes (Majkowska-Skrobek et al., 2018), and the complement system (Olszak et al., 2017; Oliveira et al., 2020). On the other hand, inflammation driven by PAMPs decreases, reducing its pro-inflammatory effects (Miernikiewicz et al., 2016; Moye et al., 2019; Pabary et al., 2016) and promoting tissue repair (Zhang et al., 2020).

Direct interactions between phages and innate immunity had also been described (Fig. 2A). Mixed pro-inflammatory and anti-inflammatory responses to various phages have been reported. For instance, certain phages in monocytes can induce the expression of pro-inflammatory cytokines such as: IL-1α, IL-1β, IL-6 and TNFα, while stimulating anti-inflammatory cytokines and regulators of pro-inflammatory cytokines, including IL-10, Interleukin 1 receptor antagonist (IL-1RA), and Suppressor of Cytokine Signaling 3 (SOSC3) (Van Belleghem et al., 2017). Other cell lines, such as intestinal epithelial cells, can be stimulated by phages and produce in response pro-inflammatory and anti-inflammatory cytokines, mainly IL-6, TNFα and IL-10, whose levels vary among phages infecting the same bacterial host (Khan Mirzaei et al., 2016). Therefore, the mixed expression of cytokines can result in a predominant anti-inflammatory effect, due to regulatory compensation driven by antagonistic cytokines.

Other studies have found mainly pro-inflammatory responses to phages, in which cytokines such as TNFα (Yıldızlı, Coral & Ayaz, 2020), IFN γ (Gogokhia et al., 2019), IL-1β, IL-6, and IL-17A (Pincus et al., 2015; Gogokhia et al., 2019) were reported. However, the extent to which phages can be considered harmful because of inflammatory-associated reactions cannot be stated. Inflammatory cytokines can be produced only in small amounts, without affecting tissues (Park, Cha & Myung, 2014), or only producing a local inflammatory response (Pincus et al., 2015).

Moreover, some studies have recorded anti-inflammatory properties associated with phages or phage-therapy. These anti-inflammatory effects were evinced in decreasing levels of pro-inflammatory cytokines or little to no impact on their production (Dufour et al., 2019; Wang et al., 2018a; Wang et al., 2018b; Freyberger et al., 2018). Besides, interactions between phages and the immune system can be different in the presence of bacteria or bacterial infection. Dufour et al. (2019) showed that 536_P1, an *Escherichia coli* phage, can induce IL-12 and IFN γ in lung tissue of healthy mice. It is noteworthy to mention that IL-12 and IFN γ are involved in the differentiation and function of Th1 cells, respectively (Sallusto, 2016). Nevertheless, in *E. coli* infected animals, pro-inflammatory cytokines declined when treated with this phage (IL-12, IL-6, IL-1β, and TNFα). These results imply that phages may modulate an initial innate response, that can be combined with a reduction of pro-inflammatory cytokines (Rouse et al., 2020). Together, the aforementioned findings describe complex immunomodulatory activities driven by phages. Indeed, it has been recorded the upregulation of IL-2R+ γ*δ* T cells by a *Brucella* phage. And, at the same time, this phage reduced TNFα produced by γδ T cells activated by bacterial LPS. Comparable results were also obtained in human macrophages (Hedges et al., 2020). IL-2 stimulate T cell growth and differentiation, and at the same time is a cytokine produced by γδ T cells that are usually found in epithelial and mucosal tissues where contribute to tissue repair and interact with innate and adaptative immunity cells (Giri & Lal, 2021; Arenas-Ramirez, Woytschak & Boyman, 2015). Collectively, these findings indicate that phages can display complex interactions that have impact on different tissues and cells.

The immune regulations displayed by phages can also modulate bacterial populations. Examples of this are Ankyphages, prophages that have a protein called ankyrin, a membrane protein associated with the cytoskeleton. It has been found that bacteria harboring these ankyrin-producing phages can evade phagocytosis, through modulation of pro-inflammatory responses driven by macrophages. This activity was related to the maintaining of certain microorganisms that possess ankyrin. More interestingly, Ankyphages can be encountered in many organisms, including the human digestive tract (Jahn et al., 2019). Hence, recognizable patterns of innate responses to phages remain to be fully identified. This immune response might be phage-specific and dependent on the responding cell. For instance, SATA-8505, a *Staphylococcus aureus* phage, can induce IFN γ production in keratinocytes or DCs, but none in peripheral blood mononuclear cells (Pincus et al., 2015).

The production of a varied repertoire of cytokines in response to phages indicates that phages are sensed by innate immunity cells. Activated macrophages can endocyte phages, possibly via a bacterial-dependent pathway (Hodyra-Stefaniak et al., 2015). Indeed, antigen-presenting cells, like DCs, can also endocyte phages, modulating responses by other immune cells. Endocytosis of the filamentous *Pseudomonas aeruginosa* phage Pf had been related to the production of type I IFN by DCs. Thus, innate IFNs production downregulated TNFα and its subsequent pro-inflammatory activities in murine phagocytes (Sweere et al., 2019). Interestingly, endocytosis of phage particles may not be restricted only to immune cells, endothelial cells could also endocyte phages. Phages can be endocyted by cerebral endothelial cells and are likely transported by the endomembrane system through Golgi apparatus, then located in vesicular and cytoplasmic compartments. These findings support phage presence in blood (Nguyen et al., 2017). Therefore, phage endocytosis by non-immune cells should also be considered and studied. Nonetheless, not all phages provoke a cellular response in phage therapy contexts: other studies did not show perturbations in the numbers of neutrophils, monocytes, lymphocytes, or eosinophils (Rouse et al., 2020; Borysowski et al., 2017). Additionally, like cytokine responses to phages, different phage strains belonging to different viral families may elicit contrasting cellular responses (Dufour et al., 2019).

### Possible pattern recognition receptors involved

Most studies that have evaluated immune responses to phages have measured final effector responses (*i.e.*, cytokines or immune cell populations); meaning that, the whole process of recognition, signal processing, and outcome is not defined or easily recognizable. In fact, available literature on phage recognition by Pattern Recognition Receptors (PRRs) is scarce and contradictory. Although phages are not considered eukaryotic parasites, it has been proposed that they can activate immune responses that are typical of eukaryotic viruses. Surveillance immune cells may detect, endocyte, and process some phages in a similar fashion to several viral pathogens. For instance, Pf *P. aeruginosa* phage RNA has been proven to trigger TLR-3 signaling in phagocytes. This finding suggests that human cells can sense phage-derived RNA. However, the possibility of having viral RNA packaged in phage capsids or RNA derived from bacteria should not be completely excluded, given that Pf is a lysogenic phage that must be induced from its host (Sweere et al., 2019). Therefore, phage sensing in this case cannot be fully confirmed. Intracellular sensing of foreign viral genomes can also be directed by another PRRs, such as RIG-I and/or MDA5. RIG-I detect short viral RNA motifs, while MDA5 sense long viral RNA motifs, producing type I and type III IFNs (Chow, Gale & Loo, 2018). Phages are not completely excluded from RIG-I sensing, given the molecules’ ability to recognize prophages, which may play a minor role in the maintenance of commensal intestinal lymphocyte populations (Liu et al., 2019). However, this assumed ability is controversial and requires confirmation. It is more likely that AIM2-Like Receptors (ALR)—which can sense intracellular invading DNA, from viruses and bacteria—may detect phage DNA (Brubaker et al., 2015).

### Adaptative immune responses

Phages can stimulate adaptative cell activity (Fig. 2B), promoting the production of specific anti-phage antibodies after phage therapy, or in experimental administration of phages to mice. In particular, Immunoglobulin G (IgG) antibodies appear to be the main humoral response to phages, detected in blood or sera (Majewska et al., 2015; Hodyra-Stefaniak et al., 2015; Rouse et al., 2020; Zaczek et al., 2016). Other antibodies were identified, such as Immunoglobulin M (IgM), that was detected in intraperitoneally injected mice with the *P. aeruginosa* phage F8 (Hodyra-Stefaniak et al., 2015), and in sera of persons treated with a *S. aureus* phage cocktail orally or locally (Zaczek et al., 2016). Immunoglobulin A (IgA) was also detected in the gut of mice orally fed with T4, an *E. coli* phage. Antibody occurrence was related to increased phage titers, and they were more prevalent after the second doses of the phage (Majewska et al., 2015). In another study, Immunoglobulin E (IgE) was slightly increased after intraperitoneal injection of an *Acinetobacter baumannii* phage cocktail, consisting in two phages: PBAB08 and PBAB25 (Cha et al., 2018). Induction of anti-phage antibodies also varied in time, from production peaking at 5 days (Rouse et al., 2020) to antibody presence up to 36–79 days, after long exposure to the phage (Majewska et al., 2015).

Briefly, antibodies can recognize phages but this recognition is variable. Despite not fully elucidated, phage recognition by antibodies may occur via immunogenic capsid proteins. Because phages have a modular genome, it is proposed that their capsid proteins might have shared motifs. Thus, some proteins from different phages exhibit a similar immune response (Van Belleghem et al., 2017). Perhaps, specific immunological responses may be developed by recognition of certain peptides, protein domains, or protein motifs, even for the same phage. Indeed, Hoc and gp12 capsid proteins, belonging to *E. coli* phage T4, are highly immunogenic whereas antibodies do not recognize other capsid proteins. The immunogenic phage proteins allow phage recognition by the circulating antibodies (Majewska et al., 2015). In fact, this phage presents Ig-like domains in proteins of its capsid that likely mediates adhesion to mucus glycoproteins present on intestinal epithelia, supporting a non-mammalian, adaptative immunity (Barr, Youle & Rohwer, 2013).

Antibody production specific for certain phages evinces the continuous presence of phages in the body. As a matter of fact, phages colonize several parts of the body, including the gastrointestinal tract, oral cavity, lungs, skin, and urinary tract. This is defined as the “phageome” and comprises almost 90% of the identified viruses’ genes from mammalian organs (Barr, 2017). Circulating anti-phage antibodies may hamper phage therapy by reducing the killing activity of the administered phages (Pincus et al., 2015) or impeding phage circulation through the body (Hodyra-Stefaniak et al., 2015; Majewska et al., 2015). However, there are some reported cases in which phages intended to be used in phage therapy are recognized by antibodies, without affecting therapy efficacy (Zaczek et al., 2016; Rouse et al., 2020). Several immunoglobulin isotypes can be produced against phages depending on their type and the route of administration. The role of these antibody responses is yet to be clarified. To the best of our knowledge, direct phage interactions with B cells are yet to be proven, despite the evidence of anti-phage antibody production.

Phage interactions with other lymphocytes have been described. Colorectal cancer-ill mice treated with phages, derived from human gastrointestinal tract or T4 phage, presented increased levels of Th cells and T cytotoxic cells (Tc) in mesenteric lymph nodes. Similar results were obtained when administering purified phages, especially for Th cells and a subpopulation of this cells, IFN γ-producing Th1 cells (Gogokhia et al., 2019). These cells are important Th-derived cells, since they play important roles in controlling intracellular pathogens by stimulating phagocytes and other cells with IFN γ (Sallusto, 2016). While expanded lymphocyte population can aid therapies in which an augmented immune response is required (*i.e.*, successful tumor control), exacerbated IFN γ-producing cells could deteriorate inflammatory status in individuals with bowel inflammatory diseases. This altered state is sometimes associated with disbalance in phage and bacteria populations (Seth et al., 2019; Maronek et al., 2020). Hence, careful investigation on phage-bacteria population dynamics should be addressed in phage therapy, in particular the effect of relevant bacteria populations or bacterial metabolites in treatment tolerance (Dery et al., 2020). Nevertheless, phages can also promote positive immune responses, by promoting microbiota balance or reducing their impact on healthy microbiota (Febvre et al., 2019). In some cases, phages were not related to increased immune cellular response. Those that do not provoke augmented pro-inflammatory cytokines, may also fail to alter immune cell populations, even from initial recognition of antigen presenting cells (Freyberger et al., 2018). Reduction of cellular immunity responses can be related to successful phage therapy and reduction of stress marker genes (Nale et al., 2020). Although phage interaction and eventual modulation of immune responses had been described, the precise mechanisms of phage-immune cell activation or stimulation remain to be understood.

As mentioned earlier, direct interaction of phages with immune receptors are not completely known. Nevertheless, some studies suggest this interaction, as outlined in this section and depicted in Fig. 2. Phage intracellular-derived peptides may be presented in the context of the Major Histocompatibility Complex (MHC) HLA class I. The processing of phage antigens and subsequent presentation by these molecules may occur via bacteria or phage endocytosis. Prophages can code proteins with epitopes that would be processed and presented by HLA class I, promoting adaptive responses by stimulating CD8+ T cells. There are even some phage-derived epitopes that are similar to tumor epitopes, thus provoking crossed reactions that result in enhanced CD8+ T cells cytotoxic response to cancer (Fluckiger et al., 2020). Given the role of HLA class I molecules in intracellular antigen processing and presentation to T cells (Rossjohn et al., 2015), phage sensing due to endocytosis and resultant immune modulation responses need further exploration.

## Perspectives for Immunomodulatory Acne Phage Therapy

To date, studies on *C. acnes* phages and acne phage therapy are scarce. The available information about *C. acnes* phages has been reviewed previously (Castillo, Nanda & Keri, 2019; Brüggemann & Lood, 2013; Jończyk-Matysiak et al., 2017) in studies that outline the history of *C. acnes* phages, phage characteristics, and prior *in vitro* studies. Below, we present a summary of their main findings.

The first isolated and characterized *Cutibacterium* phages were used to identify the *Corynebacterium* species, then classified into the *Propionibacterium* genus. Later, these early isolated phages were used to divide the genus into two morphologically related but different genera: *Corynebacterium* and *Propionibacterium*. Moreover, *Corynebacterium acnes* strains were further divided using phage’s ability to differentially infect various strains; resulting in the classification of these strains into new *Corynebacterium* species (Brüggemann & Lood, 2013). Like its host bacteria, *C. acnes* phages are usually found in the skin, in the pilosebaceous unit, and their titers are usually lower than *C. acnes* cell densities (Castillo, Nanda & Keri, 2019). The principal sources of *C. acnes* phages are sebum-rich areas, but they are isolated from other skin areas, including nostrils, oral cavity, and the gastrointestinal tract (Brüggemann & Lood, 2013). Due to the restricted *C. acnes* niche, it is thought that *C. acnes* phages are derived from a single clone that has been conserved given the low possibility of interaction with other bacterial species in this anaerobic and lipid-rich environment. This theory is supported by high nucleotide identity among *C. acnes* phage genomes, genome organization, and almost identical viral morphology, even between phages isolated from different countries (Castillo, Nanda & Keri, 2019; Brüggemann & Lood, 2013, Vives et al., 2018). Although lytic *C. acnes* phages have been described, most of those characterized are pseudolysogenic and have *Siphoviridae* morphology (Castillo, Nanda & Keri, 2019). Pseudolysogeny is an unstable life cycle presented by some phages, in which a phage exists inside the cell as an episome, outside bacterial chromosome. This state does not allow direct bacterial lysis and may conduce to bacterial resistance to other phages, by means of superinfection establishment to secure pseudolysogenic phage lifestyle (Jończyk-Matysiak et al., 2017).

Early studies on *C. acnes* phages as potential phage therapy agents were conducted *in vitro*, testing their ability to kill their host embedded in different types of formulations: ionic creams and water-oil nanoemulsions. In these preparations, phages were able to lyse bacteria and maintain their stability over several days (Jończyk-Matysiak et al., 2017). However, to date there are no *in vivo* studies in humans that explore *C. acnes* phages’ efficiency in killing this bacterium, even though various phages have been isolated and characterized from acne-affected and healthy individuals. Thus, data on phage therapy to treat acne in humans is still lacking (Castillo, Nanda & Keri, 2019).

Possible limitations to acne phage therapy have also been discussed and reviewed (Castillo, Nanda & Keri, 2019). Previous works have found that different *C. acnes* strains are sensitive to various phages, and these phages can infect diverse *C. acnes* strains. In fact, initial studies on *C. acnes* phage typing (the use of phages to identify new bacterial species based on phage ability to differentially infect certain species) become useless because of the broad host range that these phages have within the *C. acnes* species (Brüggemann & Lood, 2013). In principle, this can represent an advantage in terms of finding lytic phages for phage therapy purposes. Nevertheless, previous studies indicate that some *C. acnes* isolates can develop resistance to individual *C. acnes* phages. The use of phage cocktails is the first answer to tackle phage resistance, but it is important to further explore this approach in *C. acnes* isolates considering the phages’ limited genetic diversity (Castillo, Nanda & Keri, 2019). Lysogeny also represents a drawback since this life cycle type does not allow the rapid lysis of the bacterium (Jończyk-Matysiak et al., 2017). Thus, it is important to refine the search for *C. acnes* phages for those that only have lytic life cycles.

Besides phage therapy, phage-derived products offer additional alternatives. Endolysins are phage-encoded enzymes involved in the lysis of bacteria required for the viral progeny liberation. *C. acnes* phages encode endolysins, which are highly conserved. These enzymes catalyze the bacterial peptidoglycan destruction, and there is low reported resistance to these anti-bacterial agents (Castillo, Nanda & Keri, 2019). Another option is the concomitant use of phages and antibiotics. Their combined use can restore sensitivity to antibiotic-resistant *C. acnes* strains. In this scenario, phages establish selection pressure on resistant bacteria that present multidrug efflux pumps, which are receptors for certain phages. Consequently, phages select bacterial populations sensitive to antimicrobials (Saha & Mukherjee, 2019). Antibiotic effectivity is also achieved by using phages as sensitivity genes vectors or as antimicrobial vehicles to deliver antibiotic molecules in the site of infection. However, special care must be taken to avoid double-resistant phage bacterial isolates, by using phage cocktails or gradual use of phages and antibiotics (Jończyk-Matysiak et al., 2017). Phage cocktails should be explored while new *C. acnes* phages are found. Nevertheless, if genomes of new phages still demonstrate low genetic diversity, gradual use of single phages, custom individual therapies, and phage genetic modification would be studied.

These previous studies considered phage therapy from the most classical approach: by testing the lysis efficacy of phages. However, phages can interact with eukaryotic organisms and have a more systemic effect by modulating bacterial populations, hence impacting human microbiota, which in turn influences host health. As discussed in this review, *C. acnes* colonization-related acne pathogenesis goes beyond its abundance in affected skin, to be more closely related to a microbial dysbiosis characterized by the loss of *C. acnes* diversity. Production of short-chain fatty acids and differential immune response to *C. acnes* phylotypes can increment inflammatory responses, which is a hallmark for acne pathogenesis (O’Neill & Gallo, 2018). In fact, a high abundance of *Cutibacterium* spp. and *C. acnes* phages are related to healthy skin. In contrast, acne affected skin has lower richness of *C. acnes* phylotypes and *C. acnes* phages which has been associated to the augmented presence of virulence factors (Barnard et al., 2016). Interestingly, the presence of complete bacterial “immune” CRISPR systems has been reported in most type II *C. acnes* strains; isolates characterized into the phylotype II are usually related to healthy skin. In contrast, disease-related type I and III isolates apparently do not have entire CRISPR systems, which may have allowed a more expeditious acquisition of virulence traits to these strains (McLaughlin et al., 2019). Additionally, the absence of a competent CRISPR system in isolates belonging to phylotype I can be related to their susceptibility to lytic phages, as has been reported (Webster & Cummins, 1978). According to these findings, strains that belong to phylotype I are usually more susceptible to lytic phage infection (McLaughlin et al., 2019). This would represent a change in the classic viewpoint of selecting phages for phage therapy with a broad host range (Fernández et al., 2019), since healthy skin is characterized by a high diversity of phylotypes, in particular abundance of II and III phylotypes (Barnard et al., 2016). Therefore, selection of *C. acnes* phages should focus on those specific for strains belonging to phylotype I, aiming to restore skin microbial homeostasis and diminish exposure to virulence-bearing isolates that promote inflammation (Spittaels et al., 2021). Nonetheless, this approach must be further explored due to the limited genetic diversity of *C. acnes* phages.

Although antibiotics have anti-bacterial and immunomodulatory effects, these benefits may be limited or short-termed. Besides, these benefits are not comparable to potential long-lasting perturbations to the microbiota and development and spread of antibiotic resistance (Knackstedt, Knackstedt & Gatherwright, 2020). Phages or phage lysins can be used as selective microbiome therapeutics and contribute to restore the microbial balance in the skin (Woo & Sibley, 2020). Microbiome-based therapies may have better benefits to acne patients in re-establishing a regulated microbiota, which in turn will influence the immune response function (Xu & Li, 2019). However, the relationship between *C. acnes* and other skin microbiota organisms must be explored in greater detail. Culture-based studies on acne-related microbiota are biased because they do not reveal the complete skin microbial community (Ramasamy et al., 2019). Furthermore, there are other potential microbial agents that should be considered in acne pathogenesis. Microbial dysbiosis can also include changes in *Staphylococcus epidermidis* richness, which is suggested to be related to *C. acnes* uncontrolled colonization of the skin (Claudel et al., 2019). *Malassezia* spp. are commensal inhabitants of the skin that are related to other pathological conditions. Like *C. acnes*, this yeast can also metabolize lipids and produce irritant and allergenic molecules that compromise skin immune homeostasis (Ramasamy et al., 2019). A summary of different studies of phage therapy targeting other skin bacteria different to *C. acnes* to treat various dermatosis, including acne, was published by Steele and coworkers (Steele et al., 2020)

To the best of our knowledge, there are only two studies that addresses the relationship between *C. acnes* phages and the immune system. Kim et al. (2019) used a murine model to examine the efficacy of *C. acnes* phages in alleviating inflammatory lesions caused by *C. acnes* inoculated in mice skin. Phage administration was associated to decreased nodule size, compared with control groups. Histological findings showed reduced epidermal thickness in the phage treated group, which was in turn related to decreased expression of Integrin α6, a molecule known to cause epidermal hyperplasia. Moreover, a reduction of inflammatory markers, such as neutrophils, IL-1β, MMP-3, MMP-9 and CD8+ T cells (Kim et al., 2019), related to acne pathogenesis was observed in phage treated animals. More recently, Lam et al. (2021) carried out similar experiments to assess the therapeutic effect of a *C. acnes* phage. In this study, *C. acnes* was also inoculated on mice skin and reduction of lesions was evaluated *post-mortem*. Reduced inflammation of the lesions was observed and confirmed by histological findings. IL-1β expression in inoculated skin was reduced by phage therapy, along with the apoptosis marker Caspase-3 (Lam et al., 2021). These studies show that *C. acnes* phages can have anti-inflammatory properties. However, the mechanisms by which phages can diminish inflammatory reactions associated with *C. acnes* colonization must be further explored, especially in human cells. As reviewed earlier, these effects can be derived from bacterial elimination and subsequent control of bacterial population, or phages directly interact with certain components of the immune system, as has been reported for other phages that were associated with inflammatory response reduction. Phages may exert different immune reactions in their interaction with different cells (Khan Mirzaei et al., 2016); even different capsid proteins can represent contrasting relationships with the immune system (Dabrowska et al., 2014). Thus, selection of phages for acne phage therapy should include assessment of the relationship between candidate phages and immune response.

The most remarkable difficulty in the search for lytic phages useful in acne phage therapy is the limited diversity of phages. Nevertheless, further studies on *Cutibacterium* phage diversity are worthy. For other *Propionibacteriaceae* species, phages have been characterized with different morphologies, including prophages (latent state of a phage consisting in its genome inserted into bacterial genome) and filamentous ssDNA phages infecting *Propionibacterium freudenreichii* (Brüggemann & Lood, 2013). For that matter, a prophage found in *P. freudenreichii*, named PFR1, can cause lysis in a *C. acnes* strain by inducing the lytic cycle of a pseudolysogenic phage found in its genome. Interestingly, another *P. freudenreichii* prophage (PFR2) was able to infect *C. acnes* cells without causing bacterial lysis, becoming a prophage (Brown et al., 2017; Łoś & Wegrzyn, 2012). These findings show that prophages or lysogenic phages can interact with each other and provoke lysis, but it also indicates that phages infecting other *Propionibacteriaceae* genera may infect *C. acnes*, broadening the limited diversity of phages. Nevertheless, more studies are needed given the scarce information about lysogenic phages infecting *C. acnes*.

Acne phage therapy would be likely addressed via topical application. As a general principle, local phage therapy is easy to implement since it does not have to overcome physical and chemical barriers that diminish phage titers (*i.e.*, gastrointestinal tract). However, mechanisms of phage transcytosis through the skin require further exploration (Huh et al., 2019). *C. acnes* phage penetration should be evaluated, since most of the *C. acnes* population is inside pilosebaceous units, which can be considered a physical barrier (Christensen & Brüggemann, 2014). Moreover, hyperkeratinization in acne involves the process of comedogenesis due to abnormal keratinocyte differentiation and accumulation of lipid droplets (Kurokawa et al., 2009). Such conditions may hamper phage penetration into the skin, implying the need for excipients or pharmaceutical vehicles to improve phage absorption. Depending on the phage, conjugation with emulsions, liposomes or similar vehicles may represent a reduction of phage titers or limited phage stability (Jończyk-Matysiak et al., 2017). Moreover, phage penetration to biofilms generated by *C. acnes* IA_1_ strains should be considered (Kuehnast et al., 2018). Future studies on acne phage therapy must emphasize phage interaction with skin barrier and control of biofilm-embedded *C. acnes*, of strains belonging to IA_1_ phylotype.

## Conclusions

The paradigm of phage therapy as an alternative to antibiotic therapy is broadening to consider its additional effects. By regulating bacterial population, phages may exert indirect mechanisms that help balance the microbiota, and therefore immunity. Moreover, direct impact on immune responses is recorded for several phages. The blockage of bacterial virulence factors by phages can reduce inflammatory reactions. Other phages have been correlated with the reduction of pro-inflammatory cytokines, or the expression of anti-inflammatory cytokines. Inflammatory markers such as IL-1, IL-6, IL-8, MMPs, and TNFα are the most relevant cytokines expressed in acne pathogenesis. Since there are isolated phages that have regulatory properties on these cytokines, phage therapy studies for acne therapy should emphasize in the activity of *C. acnes* phages regarding the control of these inflammatory cytokines. The mechanisms by which cytokine regulation related to phages or phage therapy is presented is not fully known; their regulation may be given by the expression control of NF-κB or inflammasomes.

The impact of phage therapy on lymphocytes should also be evaluated. Late acne immune responses involve various Th cell lineages, including Th17, Th1, and Treg. It would also be important to evaluate the effects of any emerging therapy to treat acne in relation to the modulation of the response of these families of T cells, given the impact that these cells may have on acne inflammatory persistence and abnormal keratinization of the skin.

Phage therapy for acne and *C. acnes* regulation should be evaluated from an immunomodulatory and microbiota restoration perspective. As acne is a multifactorial disease with a markedly inflammatory nature, emerging phage therapy to manage it should have different functionalities that help to diminish or control inflammation. This skin condition involves various mechanisms of immune responses, ranging from innate immunity to cellular responses. Successful phages to treat acne might be related to the regulation in the expression of receptors such as TLR-2, TLR-4, and CD36; reduction of the activity of inflammasomes (specially NLRP3) and NF-κB; and modulation of lymphocyte and other immune cells chemoattraction by reducing KC, CXCL2, and IL-8 chemokines. Other desired effects might also be expected in acne phage therapy, such as a reduction of MMPs expression and Th cells modulation and to avoid neutralizing antibodies, which may have the potential of reducing not only inflammatory lesions but also to lower abnormal scarring risk. These immunomodulatory events in combination with the reports that indicate that phages are generally innocuous to human cells position phage therapy as a promising alternative therapy to regulate acne pathogenesis.

## Supplemental Information

10.7717/peerj.13553/supp-1 Table S1 Classification of acne severity and clinical presentation

10.7717/peerj.13553/supp-2 Table S2 Retinoid molecules used in acne treatments Chien (2018), Bell et al. (2021), Khalil et al. (2017).

## Additional Information and Declarations

### Data Availability

The following information was supplied regarding data availability: This article is a literature review.
