# Supplementary material for: The immunomodulatory potential of phage therapy to treat acne: a review on bacterial lysis and immunomodulation
Source: PeerJ. 2022 Jul 25;10:e13553. doi: 10.7717/peerj.13553 (PMC9332329; doi:10.7717/peerj.13553)
Supplement: Table S1 [file peerj-10-13553-s001.docx]

**Supplementary Table 1. Classification of acne severity and clinical presentation**

| Acne type | Severity/  Inflammation | Featured clinical presentation | Lesion type and presence in the face | | |
| --- | --- | --- | --- | --- | --- |
|  |  |  | Comedones | Papules and/or pustules | Nodules |
| Comedonal | Mild/ No inflammatory compromise | - Closed comedones comedones (skin-colored or whitish papules)  - Open comedones (keratotic plug, black or dark) | <20 | <10 | 0 |
| Papulopostular and nodular | Moderate/  Inflammation in comedones | - Inflammed comedones  - Erythematous papules | 10 to 40 | 10 to 40 | 0 to 10 |
| Nodulocystic and conglobate | Severe/ Follicular rupture leads to severe inflammation of surrounding skin | - Cysts  - Fistulae | 40 to 100 | >40 | >10 |
| Acne fulminans | Severe/ severe inflammation in surrounding skin and systemic vasculitis | - Polyporous comedones absent  - Osseous and/or arthritic involvement  - Ulceration of skin lesions  - Hemorrhagic erosions  - Association with rare and complex syndromes (PAPA, PAPASH, SAPHO, CAH) | Uncommon | >40 | >10 |

(PAPA: pyogenic sterile arthritis, pyoderma gangrenosum, and acne conglobate; PAPASH: Pyoderma gangrenosum, acne, and suppurative hidradenitis; SAPHO: Synovitis, acne, pustulosis, hyperostosis, osteitis; CAH: Congenital Adrenal Hyperplasia) (Moradi Tuchayi et al. 2015; Dessinioti and Katsambas 2017; Zaba et al. 2011; Degitz and Ochsendorf 2017)
