# Supplementary material for: The immunomodulatory potential of phage therapy to treat acne: a review on bacterial lysis and immunomodulation
Source: PeerJ. 2022 Jul 25;10:e13553. doi: 10.7717/peerj.13553 (PMC9332329; doi:10.7717/peerj.13553)
Supplement: Table S2 — Chien (2018), Bell et al. (2021), Khalil et al. (2017). [file peerj-10-13553-s002.docx]

**Supplementary Table 2. Retinoid molecules used in acne treatments (Chien 2018; Bell et al. 2021; Khalil et al. 2017).**

| Generation | Chemical structure | Unique features | Examples |
| --- | --- | --- | --- |
| First generation | Absence of aromatic radicals | Retain cyclic structure of vitamin A | Retinol, Retinaldehyde, Tretinoin, Isotretinoin, Alitretinoin |
| Second generation | Presence of one aromatic radical | Augmented bioavailability due to increased lipophilic interactions | Etretinate, Acitretin |
| Third generation | Presence of two or more aromatic radicals | Increased specificity for retinoid receptors due to structural rigidity | Adapalene, Tazarotene |
| Fourth generation | Presence of two or more aromatic radicals | Selective for RAR-γ receptors | Trifarotene |
